# Barriers to home care for terminally ill Turkish and Moroccan migrants, perceived by GPs and nurses: a survey

**DOI:** 10.1186/1472-684X-8-3

**Published:** 2009-01-26

**Authors:** Fuusje M de Graaff, Anneke L Francke

**Affiliations:** 1 Amsterdam School for Social Science Research, University of Amsterdam, Kloveniersburgwal 48, 1012 CX Amsterdam, The Netherlands; 2 Netherlands Institute for Health Services Research, PB 1568, 3500 BN Utrecht, The Netherlands

## Abstract

**Background:**

Previous qualitative research proved that relatives of elderly terminally ill Turkish and Moroccan immigrants experience several barriers to the use of Dutch professional home care. The aim of this study was to explore how general practitioners and home care nurses perceive the home care for terminally ill Turkish and Moroccan migrants and their families in the Netherlands.

**Methods:**

Questionnaires were sent to home care organizations and GPs working in areas where most of these migrants are living. 93 nurses and 78 GPs provided information about their experiences and opinions regarding home care for this group of patients. The data were analyzed by descriptive statistics.

**Results:**

GPs refer relatively few patients from these migrant groups to home care. They often find it difficult to assess the needs of these patients and their families. In 40% of the GPs' cases in which terminally ill Turkish and Moroccan migrants were not referred to home care, the GP regretted this afterwards: the patients had not received sufficient qualified care, and their informal carers had often become overburdened.

In addition, home care nurses often express dissatisfaction with the home care given to terminally ill Turkish or Moroccan patients, because of communication problems, the patients' lack of knowledge of the disease, or difficulties in making suitable appointments with the patient or with the family.

**Conclusion:**

Nurses and GPs cite chiefly similar factors influencing access to and use of home care as family members did in a previous study. However, according to GPs and nurses, the main barrier to the use of home care concerns communication problems, while relatives cited the preference for family care as the main reason for abstaining from the use of home care.

## Background

Many studies indicate that care at the end of life does not reach all patients equally: migrants for example tend to receive less end-of-life care in hospices or at home [1-7]

Moreover, when they do receive care, the care is often hampered by communication problems [8-10]. Additionally in the Netherlands, where providing care to terminal patients and their families is one of the tasks of home care organizations, care at home seems to reach relatively few migrants [11-13].

To understand the inequality in the use of care services, the history and background of immigration is relevant. Between 1965 and 1980 large groups of workers from Turkey and Morocco came to the Netherlands. Initially, they came without their families and had the intention to return to their native countries. However, since economic circumstances in these countries were not as good as in the Netherlands, many of them decided to stay and to bring their wives and children to their new country. Although the majority of male migrants in particular integrated rather easily into the lower ranges of the labor market, their integration regarding cultural aspects was less pronounced. Broadly speaking, the educational level of these migrant groups is lower than of the general population; in particular the first generation tends not to have mastered the Dutch language very well and many of them are living in deprived areas, with few contacts with people from outside their own community [14]. This might partially explain why they have less contact with Dutch home care facilities.

Less use of home care can also be explained by demographic figures, as the migrant population in general is younger than the Dutch population. However, the number of Turkish and Moroccan elderly living in the Netherlands has doubled in the last ten years: in 1996 only 15,380 Turks and 13,875 Moroccans over 55 years of age were living in the Netherlands while in 2007, 31,742 Turks and 28,109 Moroccans were counted [15]. It can therefore be expected that in the next decades more and more people within these migrant groups will develop a terminal illness.

It is likely that the care needs of terminally ill Turkish and Moroccan patients in the Netherlands will be substantial, and will differ somewhat from the needs of Dutch patients. In order to explore the needs of Turkish and Moroccan terminally ill patients and their families, we previously performed a qualitative study investigating how relatives of terminally ill Turks and Moroccans experienced Dutch home care in the terminal phase [16]. In this earlier study, experiences of families who used home care were compared with experiences of families who did not. According to the family members interviewed several factors influence access to and use of home care in the terminal phase. Some factors concerned the individual patient, such as a lack of understanding of the illness and the cause of death. Other factors related to the family situation, like decision making patterns and values and norms about care within the family. Additional factors concerned the community level, particularly the care given by community members and pressure from the community. Above all, the preference for care by family members was, in the perspective of relatives, the most important factor influencing all other variables [16] [see Additional file 1].

Although nearly all Turkish or Moroccan family members in this previous study stated that they preferred to care for their terminally ill relatives within the family, many of them suggested that general practitioners and hospital medical staff should have informed them at an earlier stage about the possibilities of receiving professional home care. These relatives also suggested that home care organizations should adapt their care supply and organization in order to make home care more useful for these target groups who often cannot express themselves easily in the Dutch language and who may have special needs because of their different cultural and religious background.

The findings of the study among Turkish and Moroccan family members prompted us to start an additional study exploring the ideas and practices of general practitioners and nurses working in professional home care organizations. For this present study the following research questions were addressed:

1. What experiences and perceptions do general practitioners and home care nurses have with regard to home care for terminally ill Turkish and Moroccan migrants and their families in the Netherlands?

2. What factors, according to them, influence the access to and use of home care in the terminal phase?

## Methods

### Terminology

In this article 'home care' means the nursing care, personal care, domestic help, health education and supply of technical aids offered by a professional home care organization to patients and their relatives at home. This implies that data derived from GPs concern the GPs' perspective on home care delivered by professional home care organizations and not the care delivered by the GPs themselves. At the time of this study (2004), home care was financed to a large extent by the Dutch government via a national insurance fund, and to a lesser extent by contributions of clients.

Home care organizations engage several home care providers, defined according to their educational level as registered (home care) nurses, certified nurse assistants and home helps. To facilitate the readability of this paper, we will use the term "nurses" for all these home care providers. Some home care organizations have teams of district nurses specialized in palliative care and/or home care technology but in the Netherlands palliative care is part of regular health care, so most clients will be cared for by educated nurses with at least three or four years of general professional education. Often these nurses cooperate with the family GP, because he is responsible for the medical care of his patients living at home. The general practitioner often refers patients to home care, although in the Netherlands a formal needs assessment by an independent assessment agency first has to be conducted before home care financed via the national insurance can be delivered.

In other countries terminally ill patients might be referred to specialist palliative care, but the approach of Dutch government is that palliative care should be part of regular health care, in most cases provided by generalists (GPs and nurses) [17]. In order to guarantee the provision of good palliative care, GPs and nurses can consult multidisciplinary specialist consulting teams of the Comprehensive Cancer Centres in the Netherlands. Moreover palliative care has become an important subject in the primary curriculum of GPs and at post-curricular courses for doctors and nurses organized for instance by the Comprehensive Cancer Centres.

The definition of Turkish or Moroccan migrants is not dependent on their formal nationality; they might have adopted the Dutch nationality. In this paper we define Turkish or Moroccan migrants as persons who live in the Netherlands but who were born in Turkey or Morocco respectively, or whose parents (or one of their parents) were born in Turkey or Morocco.

We defined a 'terminally ill client' as a client suffering from an incurable illness and having a probable life expectancy of not more than six months.

The central terms 'home care', 'terminally ill clients' and 'Turkish and Moroccan clients' were clearly defined in our questionnaires.

### Participants

In order to recruit nurses and general practitioners who have experience of home care for terminally ill Turkish and Moroccan patients we first established where these migrants are mainly living. Using data from Statistics Netherlands we identified the 30 areas with the largest number of Turkish and Moroccan inhabitants. In order to find general practitioners with experience with these migrant groups, we looked for the addresses of all GP practices located in the 30 relevant areas. As their number was far too large, we searched for addresses of practices within the urban areas with indications of social and economic deprivation in the 30 relevant areas. Because the majority of the Turkish and Moroccan population lives in these areas, we assumed that they would be registered with a practice located in these 'deprived' areas. Using data from a study of Devillé et al. [18] we identified the zip codes of the 'deprived' areas. We included all practices located in these areas and sent questionnaires to the 587 general practitioners working in these practices. General practitioners who did not respond received a reminder.

In order to find nurses with experience of these groups in the 30 relevant areas, we asked for the assistance of staff members of the 31 home care organizations who supplied home care in these 30 areas.

Eight of the 31 organizations abstained from cooperation for several reasons. These included 'no time' or 'limited experience with terminal patients within these migrant groups'. We asked the staff members to distribute questionnaires among all nurses who, according to them, would have experience with terminally ill Turkish or Moroccan patients. The staff members could get as many questionnaires as they thought they could successfully distribute.

In 23 of the 31 home care organizations, staff agreed to help us disseminate our questionnaires. The helpful staff of these 23 home care organizations had no exact information as to which of their nurses had recently cared for one or more Turkish or Moroccan terminally ill clients. They therefore made an estimation of the amount of questionnaires to be successfully distributed. Some of the organizations sent the questionnaires they could not distribute back to the researchers, whereas others may have kept the questionnaires without distributing them fully. However, we checked twice if they needed additional questionnaires to distribute. In total we sent 330 questionnaires to the cooperating organizations.

### Measures

The questionnaire focussed on characteristics of the respondent (GP or nurse) and their general experiences and perceptions regarding care for Turkish and Moroccan terminally ill patients.

The questionnaire contained three questions about the respondent (sex, age and nationality) and five questions about the work setting (amount of years in function, workload, region, function and number of Turkish or Moroccan terminally ill patients cared for). In addition, 15 questions were included about the respondents last Turkish or Moroccan terminally ill patient as well as 7 open and 37 closed questions about these patients' needs and their barriers to the use of home care, about contacts and communication with them and about the cooperation with other professionals around the care for these patients.

In some of the open questions we asked the GPs and nurses to report in detail about their experiences with their last terminally ill Turkish or Moroccan patient in the previous four years. This period seemed most appropriate for our purpose: it enabled us to include enough cases and avoid including cases whose details might be forgotten.

The open questions enabled the respondents to express their opinions and experiences in their own words, while the closed questions collected respondents' reactions to statements about for example the special needs of these patients, the possible constraints of the current home care services or of their own care, and communication with these patients. Respondents were asked to indicate their approval of these statements on a five point's scale. The statements and other questionnaire items were derived from three sources:

(a) insights from our earlier study of the experiences of Turkish and Moroccan migrants and their family members [16];

(b) insights from 'qualitative' interviews with 12 nurses responsible for the transfer from hospitals to home care and with 10 assessment agency professionals who have broad experience with patients from a Turkish or Moroccan background [19];

(c) insights from a previous literature study on health care use in relation to migrants [20].

We tested the content validity and usability of a draft questionnaire among three nurses and two general practitioners. In addition, content validity was established by discussing the draft questionnaire in the steering committee of the research project, involving eight experts with relevant scientific expertise or relevant care experiences. After these tests we performed some minor revisions, regarding choice of words, often related to the fact that the jargon used by nurses is often different from the jargon of GPs. The quantitative data were analyzed by descriptive statistics (frequencies and percentages) and differences between GPs and nurses were tested on statistical significance (using Chi-squares). The answers to the open questions in the questionnaires were qualitatively analysed by the first author by carefully reading and subsequently coding and categorising the answers based on their content.

The adapted model [see Additional file 2] is the result of our having combined central concepts resulting from qualitative and quantitative analyses and schematizing them. The scheme itself came about after talking over the interim analyses by both authors extensively and discussions with other colleagues and members of the supervising committee, following interim reporting.

## Results

### Response

We received 124 questionnaires (38% gross response) from nurses. Of this group 93 nurses had cared for one or more terminally ill Turkish or Moroccan patients in the last 4 years. The net response is therefore 28%.

We received 352 questionnaires (60% gross response) from GPs. Of this group 78 had cared for one or more terminally ill Turkish or Moroccan patients in the last 4 years, which implies a net response of 13%.

The nurses participating in the study were in general female (86/93 = 92.5%) with an average age of 43 years and they had been active in home care for an average of 11 years.

In contrast, participating GPs were more often male (56.8%) and somewhat older than the nurses (average of 49 years), and they also had a longer average professional experience (an average of 17.5 years as active GP). Table 1 displays the parts of the Netherlands where the respondents were working.

**Table 1:** Working area of participating nurses and GPs

Area	Number of GPs	Number of nurses
Amsterdam	20	5
Rotterdam	19	12
The Hague	10	11
Other parts of Western provinces (North and South Holland)	8	5
Central provinces (Utrecht, Gelderland)	12	11
Eastern province (Overijssel)	5	20
Southern provinces (North Brabant, Limburg)	4	29

Total	88	93

### Experiences with specific cases

We asked the respondents to describe in detail the background characteristics of their last Turkish or Moroccan terminally ill patient and their experiences in caring for them and their families. As a result, we got an impression of 169 cases as experienced by GPs or nurses. Two of the 171 respondents did not answer this particular question.

As table 2 shows, the majority of the patients described were males aged between 51 and 70 years. Most of them died of cancer. Other death causes listed were diabetes, stroke and COPD.

**Table 2:** Patient characteristics in both nurses' and GPs' cases

Characteristics		Nurses' Cases (in%) N = 91		GPs' Cases (in %) N = 78	
		Turkish N = 56	Moroccan N = 35	Turkish N = 39	Moroccan N = 39
Age	0–20 years	4	1	0	0
	21–50 years	20	17	10	15
	51–70 years	50	63	74	69
	> 70 years	27	17	15	15
Sex	women	42	29	23	36
	man	58	71	77	64
Use of home care	Use	100	100	59	62
	No use	0	0	41	39
Mastery of the Dutch language	Good	67	60	33	40
	Poor	33	40	67	60

In the cases presented by nurses, 60% of the Moroccan patients and 67% of the Turkish patients mastered the Dutch language. By contrast in the GPs' cases, only 40% of the terminal Moroccans and 33% of the terminal Turkish patients mastered Dutch. This might be an indication that patients who do not speak Dutch are less referred to home care. In the cases described by nurses many referrals to home care came from hospitals (45%). Only 37% came from GPs. The main reason for referral to home care in the cases described by nurses was the overburdening of informal carers (50%).

In more than half of the cases (59%) presented by GPs the family used home care, mainly (81%) after a referral by the GP. The main reason for GPs to refer to home care was their opinion that professional care was needed (72%) or that the family asked for support (40%). However, in 41% of the cases described by GPs, professional home care was not used at all. The main reason for not using home care according to the GPs was that the family wanted to care for the patient without professional help. However, in 42% of the cases where home care was not used, the GPs later regretted the decision not to refer, because the patient had not received proper care or the informal carer had become overburdened.

According to the general practitioners more than half of the relevant patients were satisfied with the home care provided. Nurses had the same impression. Reasons for not being satisfied were mainly rooted in communication problems. Patients' family members showed even greater satisfaction than patients, according to the responding GPs and nurses: in 75% of the cases described by nurses and in 64% of GP cases family members were satisfied with the home care delivered. Family members' dissatisfaction seemed to be mainly related to their conviction that terminally ill patients should be cared for by their own kin exclusively.

Yet nurses themselves were rather critical in their evaluation of the home care given. In two third of the cases the nurses mentioned some kind of trouble, often related to communication problems, or to difficulties in making suitable appointments with the patient or with the family regarding the care that had to be delivered. GPs had a more positive opinion; in 72% of their cases of terminally ill Turkish or Moroccan patients, the GPs qualified the home care as 'good'.

### General perspectives and experiences regarding these groups

Aside from the case histories regarding their last terminally ill Turkish or Moroccan patient, we asked nurses and general practitioners about their impressions and perspectives on these terminal patient groups in general.

There was large agreement between the responding nurses and GPs regarding the statement that in general Turkish and Moroccan terminally ill patients are in great need of 'coaching' by their GP. They also broadly agreed regarding the statement that these patients are in great need of good cooperation between home care nurses and informal carers (see Table 3).

**Table 3:** Perspectives of nurses and general practitioners on special needs regarding home care

In general Turkish and Moroccan terminally ill patients are in great need of:	% of nurses (n = 93)			% of GPs (n = 78)		
	Disagree	Neutral	Agree	Disagree	Neutral	agree
Coaching from GP	20	28	52	15	37	48
Information about Dutch care services provided by home care nurses	14	26	60	33	36	31
Information about Dutch care services provided by GPs	21	37	42	32	30	38
Good cooperation between home care nurses and informal carers	12	32	56	25	26	49
Coaching from home care organizations	16	28	56	45	30	25
Nursing care delivered by home care organizations	23	34	43	46	40	14
Personal care delivered by home care organizations	46	26	27	66	25	9

On some other issues there was less consensus. For example 60% of the nurses indicated that, generally speaking, Turkish and Moroccan terminally ill patients are in great need of information about the home care services in the Netherlands, while only 31% of the GPs agreed with this statement. Furthermore, 56% of the nurses compared to 25% of the GPs indicated that these patients in general are in great need of 'coaching' by home care professionals. Furthermore, 43% of the nurses and 14% of the general practitioners indicated that in general these patients are in great need of nursing care delivered by home care organizations.

### Perceptions on differences between Dutch versus Turkish or Moroccan patients

We also asked the professionals about differences between their experiences with Turkish or Moroccan patients on the one hand and with Dutch patients on the other. Many nurses (58%) and general practitioners (69%) indicated that in the case of Turkish and Moroccan patients it is more difficult to establish the home care needs of the patients and their family. It is difficult to identify what the patient wishes and what the different family members want, especially when family members are involved as translators.

### Perceptions on factors influencing access to or use of home care

Another set of statements in the questionnaire is related to our second research question: What factors, according to nurses and general practitioners, influence access to and use of home care in the terminal phase? These statements and the respondents' answers are displayed in Table 4.

**Table 4:** Perceptions of nurses and general practitioners on factors influencing access to and use of home care

Turkish and Moroccan terminally ill patients often have:	% of nurses (n = 93)			% of GPs (n = 78)		
	Disagree	Neutral	Agree	Disagree	Neutral	agree
Communication problems, which hamper them in understanding home care nurses*	5	16	79	15	21	64
Communication problems, which hamper them in organizing entry to home care*	4	22	74	28	15	57
A taboo on speaking about the terminal disease*	15	40	46	11	22	67
Different habits from Dutch patients, which hamper home care nurses in working with them in an easy way*	28	17	55	7	28	65
Problems in understanding why and to what extent they have to contribute financially in order to receive home care	6	29	65	9	32	59
Financial problems, which hamper them in paying for home care*	28	50	22	18	38	44
The fear that they will be the subject of gossip among members of their ethnic community because of their attempt to use home care	28	52	20	19	59	22

* Significant difference between nurses and general practitioners (p < 0.05)

According to both GPs and nurses, communication problems are a primary factor in hindering access to and use of home care: communication problems impede Turkish and Moroccan patients from understanding the nurses (according to 64% of the GPs and 79% of the nurses) and from organizing their entry to these Dutch services (57% and 74%). Communication problems hamper them in overcoming other factors as well.

Other factors include the perceived taboo on speaking about terminal illnesses in the Turkish and Moroccan families (76% and 46%) or special habits which impede home care nurses from working with them in an easy way (65% and 55%). Habits that may be different from Dutch patients concern, for instance, feeding and personal hygiene standards, but also the division of tasks between men and women within the family, the less openly expressed personal preferences and greater adherence to traditions within the communities.

More GPs are convinced that financial problems are at stake (44%) than nurses (22%). But both GPs and nurses (59% and 65%) agree with the statement that Moroccan and Turkish families have difficulty in understanding why and to what extent they have to pay for home care services, especially because they have no payment obligations for hospital care.

Few nurses (20%) or GPs (22%) think that fear of gossip in the Turkish or Moroccan community will prevent families from using home care. Professionals did not mention factors on the level of the community.

### Suggestions for improvement

Nearly all nurses and GPs put forward suggestions for improvement. The proposals included using more professional interpreters, learning more about culturally colored beliefs on illness and death, providing information to Turkish and Moroccan families by health educators, improving Public Relations within home care organizations and making the formal needs assessment procedures less bureaucratic. It is believed that the specific needs of the Turkish and Moroccan families are neglected in the current system of needs assessment by independent agencies, because specific needs do not fit very well into the formal needs assessment procedures.

On the basis of the respondents' information presented in this section and the above sections we refined model 1 and added the perspective of professionals to the upper part of the adapted model [see Additional file 2].

## Discussion

In this study we focused on the ideas and experiences of GPs and home care nurses with regard to home care for terminally ill Turkish and Moroccan patients.

Comparing our findings with the results of our previous study concerning the experiences of family members [16] shows many similarities, but also some differences.

We found that both professionals and family members distinguish factors related to the individual patient, the family situation, and the organizational level. However, professionals don't mention factors related to the community level and report some factors within the other levels that family members did not mention.

Another difference concerns the fact that professionals experience communication problems as a central barrier, aggravating all other problems. By contrast, family members often argue that they are used to communication problems and that they experience these problems also in other settings, for example in the hospital. The central barrier to home care is, according to family members, the preference of patients to be cared for by family members.

Both professionals and family members indicate that the situation of the family is relevant. But while professionals indicate that they sometimes feel obstructed by, for instance, the cultural habits of the Turkish and Moroccan families and the less openly expressed personal preferences, family members emphasize that professionals should take such features into account.

In addition, both professionals and family members agree that the information about and performance of the home care organizations are relevant factors. Family members indicated that proper information about the facilities of home care and good previous experiences with home care are major factors [16]. As for many Turkish and Moroccan families the GP is the principal source of information about home care, his referring performance can be crucial. But we just discovered in this study that GPs sometimes hesitate to refer to home care and that they agree significantly less than nurses with statements that Turkish and Moroccan terminally ill patients are in great need of information, nursing and coaching given by home care organizations.

One question to be raised is whether these findings are typical for the use and access of home care by *terminally ill *Turkish and Moroccan patients?

Our findings correspond with the results of studies on the care for and needs of chronically ill elderly (not particularly in the terminal phase) with a Turkish background [21-23]. These studies also point in the direction that Turkish families want to take full responsibility for the care of their patient, and that professional home care is seldom used. These studies also found that particularly daughters assume more and more responsibility for the ill relative, and that bedridden elderly often suffer because of the lack of professional care.

Another question to be raised is whether it is justified that we studied the *Turkish *and *Moroccan *target groups jointly. We recognize that there are important cultural differences between the groups of Turkish and Moroccan migrants and their families, e.g. related to their different socio-geographical roots and different languages. However, we considered it worthwhile to include both groups in our study, because both groups have some relevant common features: in the Netherlands they have a largely comparable immigration history, they are Muslims in a Christian society, they often have close family and community relations, their socioeconomic situation is not favorable and their self reported health status is often poor [11,12]. On the basis of our previous study among relatives, we had the impression that more Turkish informal carers than Moroccans had to combine their caring for the terminally ill patient with other duties like childrearing, and a formal job. Therefore, we expected that GPs and home care nurses would have different experiences regarding these two groups. However, most GPs and home care nurses could not discern differences, so we cannot make any statements on this topic on the basis of the present study.

Another limitation of this study is that we only included nurses and general practitioners that cared for at least one terminally ill Turkish or Moroccan patient over the last 4 years. This decision was made because we were interested in the perception of nurses and GPs with relevant experiences regarding these target groups. We therefore have no insight into the reasons why other nurses or GPs working in the same areas had not cared for terminally ill Turkish and Moroccan patients in the previous four years; nor do we know their views on care for these target groups.

Lastly, it might be questioned whether asking details about the last Turkish or Moroccan terminally ill client over these four years would have resulted in a significant recall bias. In our opinion, the rather detailed answers in the questionnaires and data of our previous and present studies tend to indicate that the recall bias may be small. Many respondents have cared for only one or two patients in this category up till now and said they remembered the specific care situations fairly well, and they gave lively descriptions of their experiences.

## Conclusion

This survey indicates that relatively few Turks and Moroccans are referred to home care, resulting in insufficient qualified care of patients and overburdened informal carers. The main barrier according to both GPs and nurses is the poor communication due to language problems. Differences between their statements indicate that nurses see fewer families that experience financial drawbacks and fewer families that have not mastered the Dutch language. Their impressions of the needs of the families and the possibilities of home care seem to be based on a more accessible group. GPs and nurses largely mention similar barriers as cited by family members in our previous study [16], but for relatives the main barrier is their preference for family care.

What practical implications for home care nurses or GPs can be derived from our findings? Our study indicates that professionals should realize that a sound assessment of the needs of Turkish and Moroccan patients and their families is needed. We would like to recommend that GPs should refer to home care in a rather early stage and not just in the terminal phase, since home care nurses might then more easily sort out the different perspectives and needs of the patients and the various family members. And we propose that home care organizations should facilitate nurses in terms of time, qualifications and translation services to perform their informing, coaching, nursing and physical caring tasks not only towards the patient, but also towards the various family members.

## Competing interests

The authors declare that they have no competing interests.

## Authors' contributions

FMdG conducted the study, performed the analyses and then drafted the manuscript. ALF contributed to the manuscript with her interpretations of results and comments on earlier drafts. Both authors read and approved the final manuscript.

## Pre-publication history

The pre-publication history for this paper can be accessed here:



## Supplementary Material

Additional file 1 **Model 1.** The figure shows the factors influencing access to and use of home care in the perspectives of family members (De Graaff & Francke, 2003). Click here for file

Additional file 2 **Model 2.** The figure shows the factors influencing access to and use of home care in the perspectives of professionals compared with the perspectives of family members. Click here for file
