# Supplementary material for: Barriers to home care for terminally ill Turkish and Moroccan migrants, perceived by GPs and nurses: a survey
Source: BMC Palliat Care. 2009 Jan 26;8:3. doi: 10.1186/1472-684X-8-3 (PMC2640378; doi:10.1186/1472-684X-8-3)
Supplement: Additional file 1 — Model 1. The figure shows the factors influencing access to and use of home care in the perspectives of family members (De Graaff & Francke, 2003). [file 1472-684X-8-3-S1.doc]

**Model 1**

**Factors influencing access to and use of home care in the perspectives of family members**

ACCESS TO AND USE OF HOME CARE

Lack of understanding of illness and cause of death

Family structure, decision making patterns, values and norms about care

Social environment: care by and pressure from the community

Information about and experiences with home care
