# Supplementary material for: Barriers to home care for terminally ill Turkish and Moroccan migrants, perceived by GPs and nurses: a survey
Source: BMC Palliat Care. 2009 Jan 26;8:3. doi: 10.1186/1472-684X-8-3 (PMC2640378; doi:10.1186/1472-684X-8-3)
Supplement: Additional file 2 — Model 2. The figure shows the factors influencing access to and use of home care in the perspectives of professionals compared with the perspectives of family members. [file 1472-684X-8-3-S2.doc]

**Model 2**

**Factors influencing access to and use of home care: perspectives of professionals and family members.**

*Family level*

Difficulties in establishing the family’s needs or making suitable appointments and financial constraints

*Financi

*Individual level*

Difficulties in discussing care services because of perceived taboo on speaking about the terminal disease

*Organizational level*

Limited information and complex referral procedures

Perspective of…………………………………………………………………………….... professionals

ACCESS TO AND USE OF HOME CARE

Perspective of………………………………………………………………………..........family members

*Individual level*

Lack of understanding of illness and cause of death

*Organizational level*

Information about and experiences with home care

*Family level*

Care given by the family depends on family structure, decision making patterns, values and standards about care

*Community level*

Care by and pressure from the community

.
